# Effect of bone quality and quantity on the primary stability of dental implants in a simulated bicortical placement

**DOI:** 10.1007/s00784-020-03432-z

**Published:** 2020-07-10

**Authors:** Stefan Rues, Marc Schmitter, Stefanie Kappel, Robert Sonntag, Jan Philippe Kretzer, Jan Nadorf

**Affiliations:** 1 Department of Prosthodontics, University of Heidelberg, Im Neuenheimer Feld 400, 69120 Heidelberg, Germany; 2 Department of Prosthodontics, University of Würzburg, Pleicherwall 2, 97070 Würzburg, Germany; 3 Laboratory of Biomechanics and Implant Research, Clinic for Orthopedics and Trauma Surgery, University Hospital Heidelberg, Schlierbacher Landstraße 200a, 69118 Heidelberg, Germany

**Keywords:** Dental implants, Primary stability, Sinus floor elevation, Bone density, Remaining bone height, In vitro simulation

## Abstract

**Objectives:**

Conventional dental implants inserted in the molar region of the maxilla will reach into the sinus maxillaris when alveolar ridge height is limited. When surgery is performed without prior augmentation of the sinus floor, primary stability of the implant is important for successful osseointegration. This study aimed at identifying the impact of bone quality and quantity at the implantation site on primary implant stability of a simulated bicortical placement.

**Materials and methods:**

In our in vitro measurements, bone mineral density, total bone thickness and overall cortical bone thickness were assessed by micro-computed tomography (μCT) of pig scapulae, which resembled well the bicortical situation found in human patients. Dental implants were inserted, and micromotion between bone and implant was measured while loading the implant with an axial torque.

**Results:**

The main findings were that primary implant stability did not depend on total bone thickness but tended to increase with either increasing bone mineral density or overall cortical bone thickness.

**Clinical relevance:**

Limited bone height in the maxilla is a major problem when planning dental implants. To overcome this problem, several approaches, e.g. external or internal sinus floor elevation, have been established. When planning the insertion of a dental implant an important aspect is the primary stability which can be expected. With other factors, the dimensions of the cortical bone might be relevant in this context. It would, therefore, be helpful to define the minimum thickness of cortical bone required to achieve sufficient primary stability, thus avoiding additional surgical intervention.

## Introduction

**Fig. 1:**
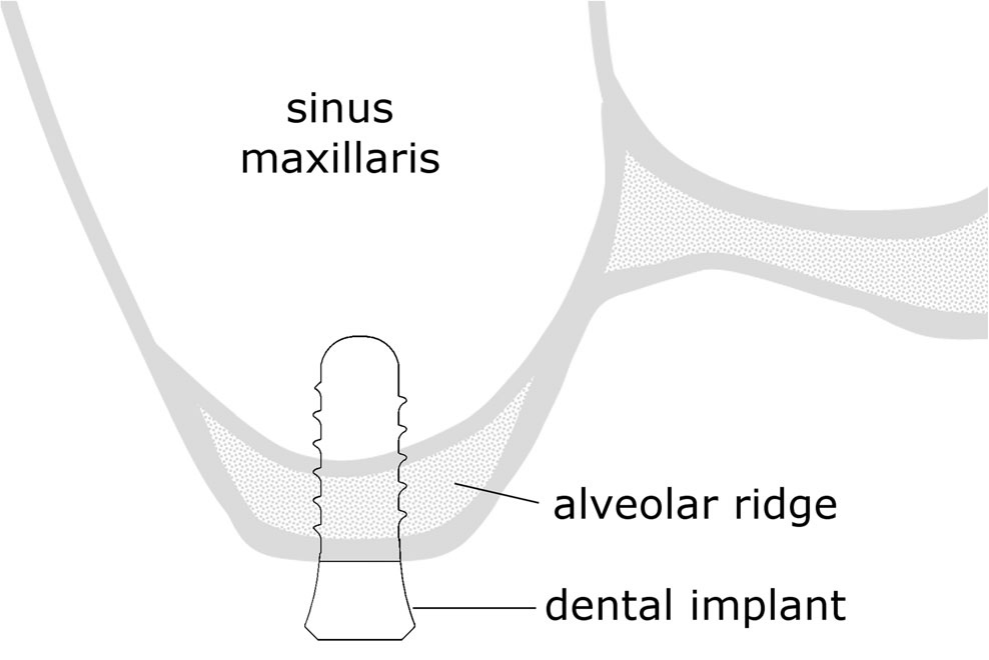
Schematic diagram of an implant inserted in the molar region of an atrophied maxilla. Without augmentation, the remaining alveolar ridge height is usually less than the length of the implant, leading to bicortical anchorage.

In recent decades, implant-supported dentures have become increasingly common in dental practice. As a result of improvement of the implant surface, excellent osseointegration rates have been achieved. In addition to the structure of the implant surface, however, the complex osseointegration process [1] is affected by other factors, e.g. primary implant stability, described as the absence of implant movement relative to the surrounding bone immediately after insertion. This, however, is affected by bone density, implant geometry, surgical technique (especially site preparation) and bone quantity [2, 3]. In the posterior region, especially, of the maxilla and mandible, bone quantity might be limited. In the maxilla, the dimension of the sinus maxillaris is the factor limiting bone quantity (cf. Fig. 1). The height of the alveolar ridge in this region is affected by numerous factors: tooth loss [4], traumatic extractions, antagonistic teeth, etc. [5]. If the height of the alveolar ridge in the maxilla is insufficient, several approaches can, nevertheless, be used to place dental implants: use of short implants [6], bone augmentation (including direct or indirect sinus floor elevation [7]) and internal sinus lift without graft material [8]. To decide which technique should be used, however, it would be helpful to know the cut-off limit of the height of the alveolar ridge enabling insertion of the implant with sufficient primary stability without augmentation. Although primary stability can be assessed in vitro by use of several approaches, for example an ultrasonic method [9], percussion testing [10] and resonance frequency analysis [11, 12], these approaches cannot be used to measure the breakaway torque of the implants, which is an important aspect of primary stability. Resonance frequency analysis, furthermore, furnishes the effective flexural stiffness of the implant–bone system, which, when bone quantity is limited, is substantially affected—even for perfectly osseointegrated implants—by the height of the alveolar ridge. Furthermore, it has been shown that insertion torque and resonance frequency analysis are independent and incomparable methods of measuring primary stability [13].

Thus, in vitro measurement of the breakaway torque and the relative movement between bone and implant while applying torsion to the implant were used in this study to assess the dependence of primary implant stability on both bone height and bone density. This test method has hitherto been used solely for research on orthopaedic implants [14, 15].

## Materials and methods

To analyse primary stability, dental implants were implanted in pig bones and subsequently loaded torsionally relative to the implant axis. During torsional load application, three-dimensional motion of markers placed on bone and implant was measured and subsequently analysed to enable quantification of implant–bone relative micromotion. Relative micromotion, implantation torque and bone quality and quantity might enable conclusions to be reached on primary implant stability.

### Specimen preparation

**Fig. 2:**
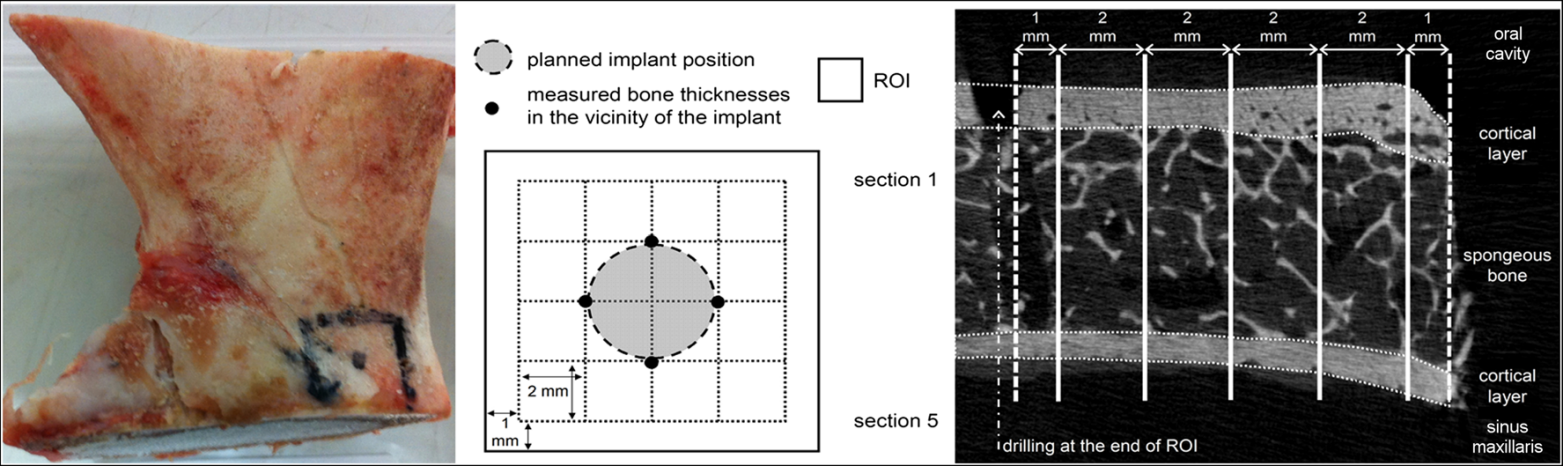
Resected pig scapulae with marked ROI (left), position of evaluation sites (grid) for thickness measurements within the ROI (centre) and bone thickness measurements in the chosen sections on the basis of μCT data (right)

Seventeen bone segments were resected from ten freshly frozen pig scapulae, providing a situation similar to that in the sinus area of human patients. A maximum segment size of 40 mm × 60 mm and a minimum segment thickness of 4 mm were chosen as the basis for further preparations. On each bone segment, a 10 mm × 10 mm region of interest (ROI) was marked with one side located at a cut edge (cf. Fig. 2, left). The position of each ROI was chosen, if possible, such that bone thickness did not exceed approximately 8 mm. To enable location of the ROI during micro-computed tomography (μCT), edges were marked with small (1-mm) drilled holes.

### Micro-CT analysis

Before implantation, three-dimensional images of the bone segments were obtained by use of X-ray μCT (SkyScan 1076, SkyScann.v.; Aartselaar, Belgium). Serial 180° imaging circulating in 0.4° steps around the longitudinal axis, with settings 80 kV, 120 μA and 460 ms, resulted in resolution of 18 μm. To enable bone mineral density (BMD) analysis, calibration objects with known BMD were also scanned. The sample-based assessment of cortical and spongeous bone structures and bone mineral density should enable a meaningful determination of the correlation of implant–bone relative motion with implant loading.

Bone mineral density and the thicknesses of the upper and lower cortical layers and the spongeous bone tissue were therefore measured within the ROI. For measurement of bone thickness, five cross-sections 2-mm apart were determined within the ROI, starting 1 mm from the edges of the ROI (Fig. 2).

The bone thickness in the vicinity of the implant (implant radius 2.05 mm) was of special interest and was given as the mean value of measurements at the four relevant evaluation sites (cf. Fig. 2, centre). These values were subsequently used to determine correlations between bone thickness and primary stability. One experienced examiner marked regions defining cortical bone and spongeous bone such that, in addition to total bone thickness, thickness of cortical layers and thickness of spongeous bone could also be extracted (Fig. 2, right).

The grayscale images (range 0 to 255) were converted to monochrome images by using thresholds [65,245] to define hard tissue (bone); grey values < 65 were indicative of soft tissue and values > 245 were associated with artefacts from the μCT scan. Mean BMD values for the hard tissue within the ROI were extracted, as also was the percentage bone volume, on the basis of the complete tissue volume for both cortical layers and spongeous bone.

### Implantation and connection to the torsion device

**Fig. 3:**
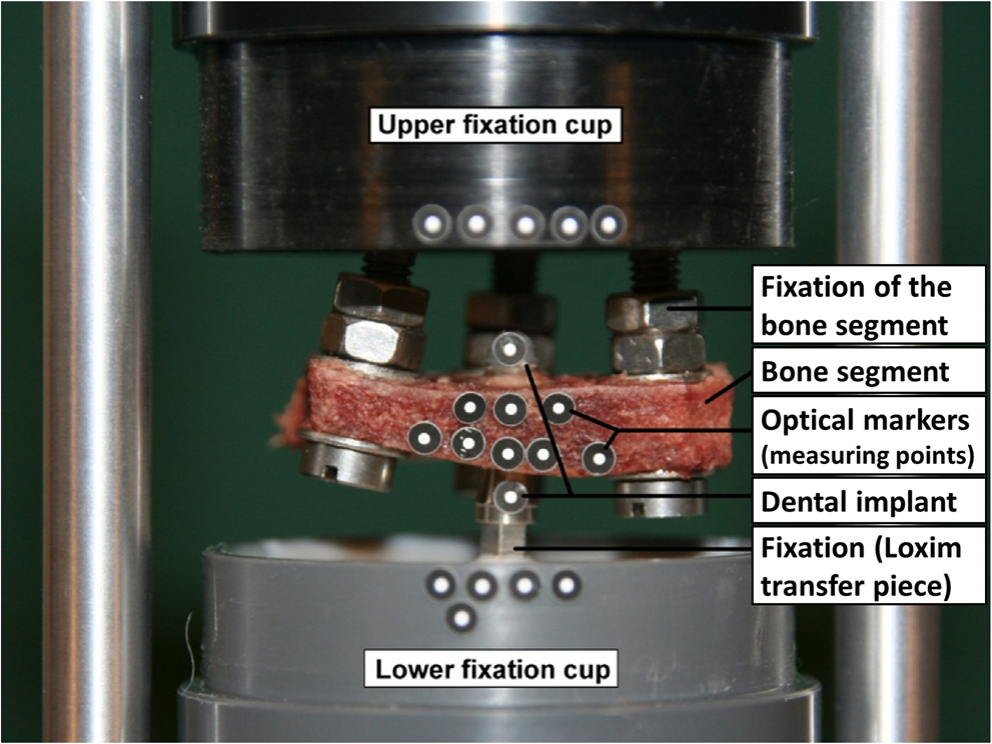
Experimental setup with inserted implant (fixed in the lower cup), bone (fixed in the upper cup) and optical markers indicating measurement points

Before implantation, each bone was fixed on one side to the upper fixing cup by use of three screws and two-component polyurethane (Fig. 3). During this process, careful attention was paid to positioning of the ROI at the centre of subsequent axial load applications.

In accordance with the manufacturer’s guidelines, *n* = 17 dental soft tissue level implants (Standard Tissue Level Implant, 4.1 mm RN, SLA®, 10 mm length; Straumann AG, Basel, Switzerland) were inserted at the centre of each ROI (Fig. 2) after preparation of the bone cavity (pilot drilling, 2.8 mm diameter and 10 mm depth, followed by 3.5 mm diameter pilot drilling just pushing through the coronal cortical layer).

Implant design can be divided into two main parts: the machined and tulip-shaped neck (2.8 mm) which is polished and which is placed within the soft tissue to enable adaption to abutments by use of the inner Morse taper and the lower 10 mm cylindrical part of the implant which should provide primary (screw design) stability after insertion in the bone tissue and secondary (macroscopic and microscopic SLA® surface modification) stability after osseointegration. A torque wrench (ratchet) was used for placement of the implants with an insertion depth of 10 mm. Hence, as in clinical practice, the torque required for insertion could differ from the ideal value of 35 Ncm suggested by the manufacturer of the implant. For each sample, the actual maximum implantation torque was documented (intervals given by the torque wrench scale: *M* ≤ 15 Ncm; 15 Ncm < *M* ≤ 25 Ncm; 25 Ncm < *M* ≤ 35 Ncm). After implantation, the Loxim transfer piece was not removed but used to fix the implant with polyurethane in the centre of the lower fixing cup, by use of a template (Fig. 3).

### Torsion device

**Fig. 4:**
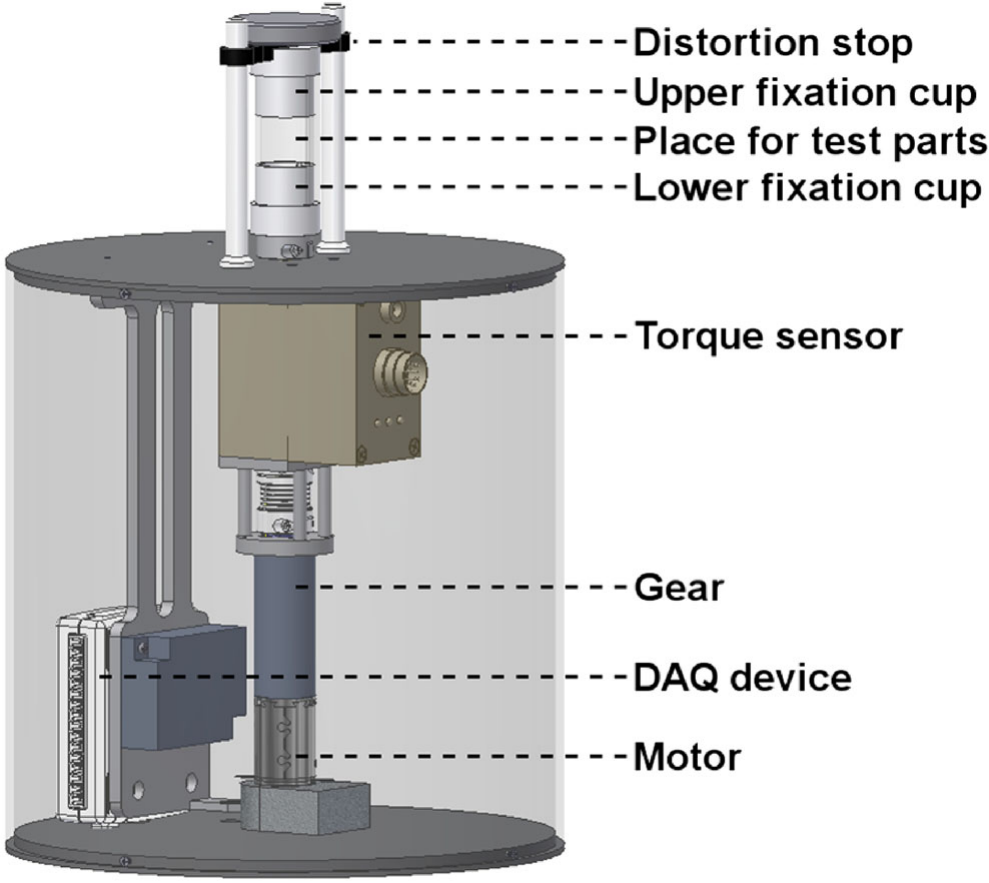
Torque measurement setup

The custom-made torque measurement setup consisted of an internal part, including torque generation and torque measurement, and an external part for positioning of the test specimens (Fig. 4). The torque control and data-acquisition software was created in the LabWindows™/CVI™ 2009 environment (Version 9.1.0; National Instruments Corporation, Austin, TX, USA). The whole test setup was controlled by use of a data-acquisition device (NI DAQ USB-6009; National Instruments Corporation, Austin, TX, USA). A DC motor including electronic and planetary gearhead (A-max-22 110130 with planetary gearhead GP-22-A 134188; MaxonMotor AG, Sachseln, Switzerland) was used to generate the corresponding torques. Applied torques were monitored by use of a high-precision torque sensor (model 8661; Burster Praezisionsmesstechnik GmbH&Co Kg, Gernsbach, Germany) with a measurement range from 0 to 50 Ncm and a rotational angle resolution of 1024 increments/0.088°. Implants were fixed between a lower, torque-applying, fixing cup and an upper fixing cup which was combined with a distortion stop.

### Settings and data acquisition

Three cycles each of ± 15 Ncm (43% of the ideal insertion torque) were applied with a rotating speed of 2°/min and with the starting direction rotating counter-clockwise. To track motion, optical markers defining specific measurement points were attached to each component (Fig. 3): five markers were fixed on each fixing cup, and each one marker was fixed at the implant neck and at the distal end (if available). Eight markers were fixed along the cortical layers of the bone segment. Since the geometry of each sample was unique and markers were placed by hand, their positioning was similar for all samples but not identical. An optical 3D measurement system (PONTOS 5 M; GOM GmbH, Braunschweig, Germany) was used to track the spatial motion of each labelled measurement point given as centre points of standardized optical markers (uncoded passive white markers, 0.4 mm in diameter, GOM Item Number: 21875; GOM GmbH) and, thus, the motion of each component during torque application, with a resolution of ± 1 μm. In literature, the accuracy of this measurement system was reported to be < 5 μm [16]. Images were generated with a frame rate of 2 Hz by using two cameras with 50 mm objectives.

**Fig. 5:**
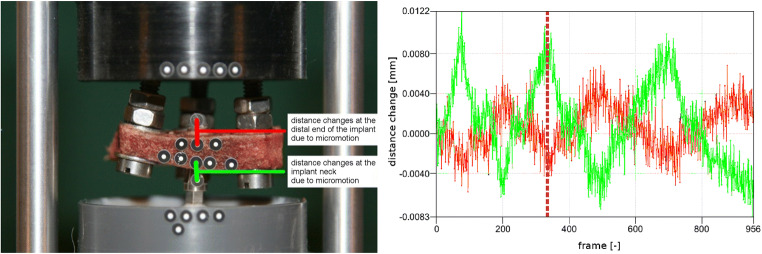
Test setup and pairs of measurement points (red: pair of measurement points at the distal end of the implant, green: pair of measurement points at the implant neck) to identify relative micromotion between bone and implant (left). Plot of relative distance changes for these two pairs of measurement points for three load cycles with maximum torque ± 15 Ncm (right)

If at least three non-collinearly placed measurement points were available on a component, its rigid body motion could be computed. If all the components were tracked separately, relative movement between any two components could be evaluated. In this work, this was achieved for all components except the implant to which only a maximum of two optical markers could be attached. Therefore, relative movement between bone and implant was evaluated by means of distance changes for pairs of measurement points (cf. Fig. 5). For additional control of the accuracy of measurement of length, distance changes within the implant were also evaluated. For the rather small torques used in the tests, elastic deformation within the implant can be neglected, i.e. the implant can be idealized as a rigid body, implying the absence of distance changes between measurement points on the implant.

To reduce noise, a sliding average (actual frame ± two frames) was computed and extreme distance changes, i.e. distance changes between torques of + 15 Ncm and – 15 Ncm, were evaluated.

Finally, mean values for all pairs of measurement points (two pairs for bone-implant micromotion) and loading replicates (three cycles) were extracted and used for statistical analysis.

### Statistics

Descriptive statistics were used, and the effects of cortical bone thickness, total bone thickness and BMD on bone-implant micromotion (given by distance changes) were analysed (IBM SPSS Statistics 21) by means of linear regression with a significance level of *α* = 0.05.

## Results

Distance changes within the implant, which can be regarded as the accuracy of this measurement method, were 1.0 ± 0.3 μm. No unintentional relative motion occurred between fixed system components, i.e. all test runs were valid.

With the exception of one sample which was not anchored bicortically, implant lengths embedded in cortical bone ranged from 1.8 to 2.5 mm (cf. Table 1). For five samples, the torsion tests could not be completed as planned because the implants could not withstand 15 Ncm torque, i.e. they loosened before reaching maximum torque. This was not unexpected, because for four of these samples, the insertion torque was below 15 Ncm and one sample was implemented with a torque below 25 Ncm. Furthermore, these samples had either less than 2 mm implant length embedded in cortical bone or the BMD was low. For normal samples, distance changes corresponding to bone-implant micromotion could be evaluated.

**Table 1:** Results from μCT data extraction and measurement of bone-implant micromotion during torsion tests (absolute values pooled from both evaluated distances)

Insertion torque	Overall implant length embedded in cortical bone	Total bone thickness	Max. torque in tests	BMD	Bone volume/tissue volume			Bone-implant micromotion (distance changes)
					Upper cortical bone layer	Lower cortical bone layer	Spongy bone	
(Ncm)	(mm)	(mm)	(Ncm)	(g/cm^3^)	(%)	(%)	(%)	(μm)
*M* ≤ 15	1.1*	12.0	12.0	1.07	89	78	18	–
*M* ≤ 15	1.9	5.6	14.8	1.15	93	94	24	–
	1.9	4.6	8.5	0.70	85	88	22	–
	2.1	5.8	6.8	1.07	93	79	21	–
15 ≤ *M* ≤ 25	1.9	4.9	9.9	1.20	82	85	26	–
	1.9	7.8	15.0	0.99	76	70	35	9.9
	1.9	5.0	15.0	1.14	90	88	25	11.4
*M* > 25	1.8	6.8	15.0	1.28	96	99	18	4.3
	1.8	4.4	15.0	1.20	84	85	29	3.5
	1.9	6.3	15.0	1.14	92	95	46	8.2
	2	6.2	15.0	1.18	85	74	27	5.6
	2.1	5.8	15.0	0.94	68	65	29	6.9
	2.1	5.1	15.0	1.14	88	89	27	4.2
	2.2	6.1	15.0	1.08	89	69	29	4.4
	2.3	8.3	15.0	1.11	87	94	18	4.7
	2.4	5.9	15.0	1.18	89	79	29	3.6
	2.5	4.7	15.0	1.14	95	91	52	3.7

*Implant anchored solely in the upper cortical layer

Distance changes caused by bone-implant micromotion showed the largest magnitudes when choosing measurement points on the bone closest to the implant axis and evaluation of other bone-implant point pairs gave similar distance changes (with smaller magnitudes). Therefore, data evaluation was reduced to the two (3-dimensional) distances indicated in Fig. 4. Since similar results with opposing sign were observed for these two distances, absolute values were pooled (→ one final measurement value for each sample, cf. Table 1).

**Fig. 6:**
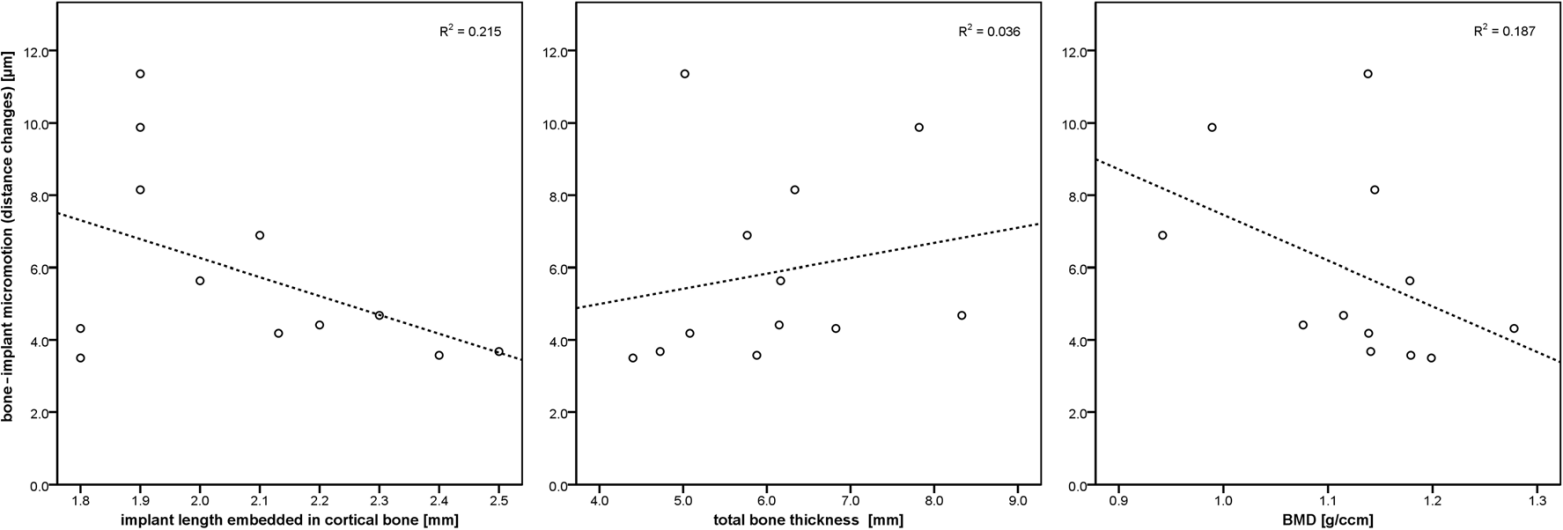
Measured data and fitted straight lines for dependence of bone-implant micromotion on implant length embedded in cortical bone (left), total bone thickness (centre) and BMD (right)

Bone-implant micromotion caused by torsion after implant insertion (cf. Fig. 6, Table 2) did not correlate with total bone thickness (*p* = 0.827) whereas bone-implant micromotion tended to decrease with either increasing BMD (*p* = 0.095) or increasing implant length embedded in cortical bone (*p* = 0.071). The lowest registered micromotion was approximately 4 μm and was usually observed for samples with more than 2.0 mm implant length embedded in cortical bone with a BMD > 1.0 g/cm^3^.

**Table 2:** Linear regression results for parameters affecting bone-implant micromotion

**Coefficients**^**a**^						
Model		Unstandardized coefficients		Standardized coefficients	*t*	Sig.
		*B*	Std. error	Beta		
1	(Constant)	34.251	12.695		2.698	0.027
	BMD (g/ccm)	− 14.696	7.765	− 0.503	− 1.893	0.095
	Total bone thickness (mm)	0.132	0.583	0.059	0.226	0.827
	Overall implant length embedded in cortical bone (mm)	− 6.082	2.922	− 0.539	− 2.082	0.071

^a^Dependent variable: bone-implant micromotion (μm)

## Discussion

Insertion of dental implants in the posterior region of the maxilla is often challenging because of the dimensions of the sinus maxillaris [17]. An increasing number of patients desire implants in this region while simultaneously declining bone augmentation. Moreover, all surgical augmentation techniques for the edentulous maxilla are prone to resorption [18].

This in vitro study was conducted to assess the effects of several factors, including bone quantity, quality and density [19] on the primary stability of implants (measured as bone-implant micromotion). This topic is of major interest, as the impact of these variables on successful osseointegration of dental implants is important for the clinician [20]. Especially, information about bone quantity could be especially helpful for the clinician attempting to decide whether implant insertion without augmentation has any chance of success in respect of the primary stability of the implant. The role of primary stability has been addressed in numerous studies, assessing several variables. However, a review published in 2017 by Greenstein & Cavallaro [21] summarized that “increased insertion torque helps achieve primary stability by reducing implant micromotion”. Thus, a higher insertion torque is a quasi-surrogate criterion for low micromotion. However, primary stability is not the premise for osseointegration, as a low micromotion might be achieved by other precautions [22].

Bone quantity in the posterior maxilla has been categorised into three groups [23]: in class 1, the residual bone height is at least 10 mm; in class 2, residual bone height is 5–10 mm; and in class 3, bone height is 0–5 mm. It has been proposed that for classes 2 and 3, sinus augmentation should be performed [17]. In recent years, however, it has been reported that implants can be placed successfully for lower bone heights [6, 24]. Thus, biomechanically, the question which arises is how much bone is the “minimum” requirement for placement of implants in this region without augmentation [25].

Because primary implant stability is affected by several factors, bone density, thickness of the cortical bone and height of the alveolar ridge were measured in this study. The results showed that bone density is likely to affect primary implant stability, which is in accordance with the results of Pommer et al. [26] and de Elio Oliveros et al. [27]. It should, however, be noted that although observed BMD ranged between 0.9 and 1.3 g/cm^3^, for most of the samples, BMD was in the range 1.1–1.2 g/cm^3^. Furthermore, the effect of the total height of the alveolar ridge had no major effect on primary stability, which is also in accordance with Pommer et al. [26]. This could be because, for our specimens, the percentage of hard tissue in the spongeous bone was low (28 ± 9%, Table 1). Since similar or even smaller values are reported for edentulous patients in the literature [28], it should be possible to correlate this finding with dental practice.

In addition to total bone thickness, the overall thickness of cortical bone along the implant was also assessed in this study and revealed a relevant effect of this on primary implant stability. This finding is in accordance with the results of Hsu et al. [29]. In our specimens the percentage of hard tissue in cortical bone was 85 ± 9% (Table 1), which is also very similar to the situation in the edentulous maxilla of human patients [28]. A study analysing the bone quality of atrophic edentulous mandibles found mean cortical bone porosity was approximately 8% [30], indicating that the porosity of cortical bone is insensitive to atrophy.

For all samples with both a BMD greater than 1.0 g/cm^3^ and overall cortical bone thickness of at least 2.1 mm, bone-implant micromotion was approximately 4 μm. This value seemed indicative of a lower threshold. We thus assumed optimum primary stability for implants in these specimens. In contrast, greater bone-implant micromotion, i.e. low primary stability, was observed for implants inserted in specimens with either a BMD less than 1.0 g/cm^3^ or overall cortical bone thickness not exceeding 2.0 mm. This includes implants for which micromotion tests failed because the insertion torque was very low. The torque magnitude of 15 Ncm (43% of the optimum insertion torque) which an inserted implant had to withstand was defined before the tests were conducted. Because this type of measurement has not hitherto been performed with dental implants, this minimum required torque resistance could not be obtained from the literature but was estimated on the basis of the experience of dental surgeons. The results suggest this choice was reasonable.

Because relative bone-implant micromotion results, depending on torque direction, in a small amount of either screw insertion or screw extraction, distance changes for pairs of promixal and distal measurement points were bound to have opposite signs (Fig. 5, right). Furthermore, distance changes within the implant, which can be idealized as a rigid body, were found to be 1.0 ± 0.3 μm, which is similar to the accuracy given by the manufacturer for the optical measurement device used and even better than the upper limit of 5 μm reported in literature [16]. Hence, measurement errors involved in our tests did not severely affect the results.

A study comparing the outer geometry of the implants [31] found that implants with conical shape had superior primary stability compared to those with a parallel shape. In our investigation, only one implant type was used. Therefore, effects of the implant geometry were not assessed and a transfer to other implant systems has to be done with care.

The use of other measurement techniques such as strain gauges and intraoral scanning devices would not have been feasible for our test setup. Strain gauges [32] would only detect (elastic) deformation within the substrate below the sensor but not relative motion at the bone-implant interface. Intraoral scanning devices, on the other hand, do not possess a sufficient accuracy to assess displacements < 10 μm [33] and the required scanning times would require load steps with constant torques during which there would be the risk of ongoing micromotion between bone and implant.

Many studies use FE analyses as a helpful tool to assess phenomenological behaviour of a mechanical problem and study the effect of controlled parameter and geometry changes [32, 34]. Since many parameters (adhesion and coefficient of friction at the bone-implant interface) would have had to be guessed and imperfections in reverse engineering would have affected the prestressed state which is essential for primary implant stability, FE simulations would not have been reliable.

## Conclusions

Within the limitations of this study, it can be concluded that total bone thickness is not a reliable indicator of primary implant stability, which seems to increase with increasing BMD and overall cortical bone thickness. For our specimens, optimum primary stability was observed for BMD > 1.0 g/cm^3^ and overall cortical bone thickness > 2.0 mm.
